# Silent polymorphisms in the *RYR1* gene do not modify the phenotype of the p.4898 I>T pathogenic mutation in central core disease: a case report

**DOI:** 10.1186/1756-0500-7-487

**Published:** 2014-08-01

**Authors:** Thais Cuperman, Stephanie A Fernandes, Naila CV Lourenço, Lydia U Yamamoto, Helga CA Silva, Rita CM Pavanello, Guilherme L Yamamoto, Mayana Zatz, Acary SB Oliveira, Mariz Vainzof

**Affiliations:** 1 Laboratory of Muscle Proteins and Comparative Histopathology, Human Genome Research Center, Biosciences Institute, University of São Paulo, R. do Matão, 106 – Cidade Universitária, São Paulo, SP CEP 05508-900, Brazil; 2 Department of Neurology and Neurosurgery, Division of Neuromuscular Disorders, Federal University of São Paulo (Unifesp), São Paulo SP, Brazil

**Keywords:** Congenital myopathies, Central core disease, Ryanodin receptor 1 gene (*RYR1*), Modifying effect, Genotype/phenotype correlations

## Abstract

**Background:**

Central core disease is a congenital myopathy, characterized by presence of central core-like areas in muscle fibers. Patients have mild or moderate weakness, hypotonia and motor developmental delay. The disease is caused by mutations in the human ryanodine receptor gene (*RYR1*), which encodes a calcium-release channel. Since the *RYR1* gene is huge, containing 106 exons, mutation screening has been limited to three ‘hot spots’, with particular attention to the C-terminal region. Recent next- generation sequencing methods are now identifying multiple numbers of variants in patients, in which interpretation and phenotype prevision is difficult.

**Case presentation:**

In a Brazilian Caucasian family, clinical, histopathological and molecular analysis identified a new case of central core disease in a 48-year female. Sanger sequencing of the C-terminal region of the *RYR1* gene identified two different missense mutations: c.14256 A > C polymorphism in exon 98 and c.14693 T > C in exon 102, which have already been described as pathogenic. Trans-position of the 2 mutations was confirmed because patient’s daughter, mother and sister carried only the exon 98’s mutation, a synonymous variant that was subsequently found in the frequency of 013–0,05 of alleles. Further next generation sequencing study of the whole *RYR1* gene in the patient revealed the presence of additional 5 common silent polymorphisms in homozygosis and 8 polymorphisms in heterozygosis.

**Conclusions:**

Considering that patient’s relatives showed no pathologic phenotype, and the phenotype presented by the patient is within the range observed in other central core disease patients with the same mutation, it was concluded that the c.14256 A > C polymorphism alone is not responsible for disease, and the associated additional silent polymorphisms are not acting as modifiers of the primary pathogenic mutation in the affected patient. The case described above illustrates the present reality where new methods for wide genome screening are becoming more accessible and able to identify a great variety of mutations and polymorphisms of unknown function in patients and their families.

## Background

Core myopathies is a group of clinically and genetically heterogeneous congenital myopathies with focally reduced oxidative activity visualized on muscle biopsy as their common defining histopathological feature [1]. These areas, defined as *cores,* can be single or multiple, central or eccentric and run through a large area by longitudinal muscle fiber axis visualized on muscle biopsy [2]. The wide range of described terms applied to these disorders (central cores, minicores, multicores, multi-minicores) is due to variability of oxidative stain irregularities seen in the histopathological analysis [3].

Central core disease (CCD, Online Mendelian Inheritance of Man #117000) is characterized by the presence of a central and single core-like area on oxidative stains [3]. It indicates areas of sarcomeric disorganization and electron microscopy displays mitochondrial depletion and variable degrees of degeneration of the contractile apparatus. Abnormal expression of several sarcomeric and intermediate filament proteins can be seen by immunohistochemical studies [4-6].

CCD is intimately linked to malignant hyperthermia susceptibility (MHS, Online Mendelian Inheritance of Man #145600) [7], a skeletal muscle’s pharmacogenetic disorder that is triggered by administration of inhalational anesthetics and depolarizing muscle relaxants in susceptible individuals [7,8].

Both conditions have been associated to mutations in human *RYR1* gene (Online Mendelian Inheritance of Man #180901), located on chromosome 19q13.1. It is a large gene organized in 106 exons [9] that encodes a calcium (Ca^2+^) release channel known as the ryanodine receptor (*RYR1*) situated on skeletal muscle. The ryanodine receptor is a huge protein of 5037 amino acids [10] constituted of four *RYR1* 560 kDa peptides and many associated proteins with a total molecular weight of more than 2,500 kDa.

Currently, more than 200 mutations in *RYR1* gene have been described. Some are exclusively associated with CCD or other *RYR1*-related myopathies; others are related with malignant hyperthermia susceptibility trait, or even with both conditions concomitantly [3].

The *RYR1* mutational spectrum linked with CCD includes mostly heterozygous dominant missense variants and minor deletions or duplications [10-13]. *RYR1* mutations associated with malignant hyperthermia (MH) and CCD are grouped in three regions of *RYR1* protein or ‘hot spots’: N-terminal (residues p.M1-R614), central (p.R2163–p.R2458) and C-terminal (p.R4136-p.P4973). These regions are also entitled domains 1–3, respectively [14]. Domains 1 and 2 are situated in soluble cytoplasmic regions of the protein. Domain 3 is placed in C-terminus and involves the transmembrane/luminal and pore-forming area of the channel. It is also where most of CCD mutations are located [11,15]. Variable genotype–phenotype correlations associated with mutations in *RYR1* gene are usually justified by the degree of functional variation within this large protein [3].

Here we present clinical features of a family diagnosed with central core myopathy, in which the affected index case, in addition to the causative mutation in the *RYR1* gene, also presented several other polymorphisms in this gene, which apparently are not acting as modifiers of the primary pathogenic mutation.

## Case presentation

**Figure 1:**
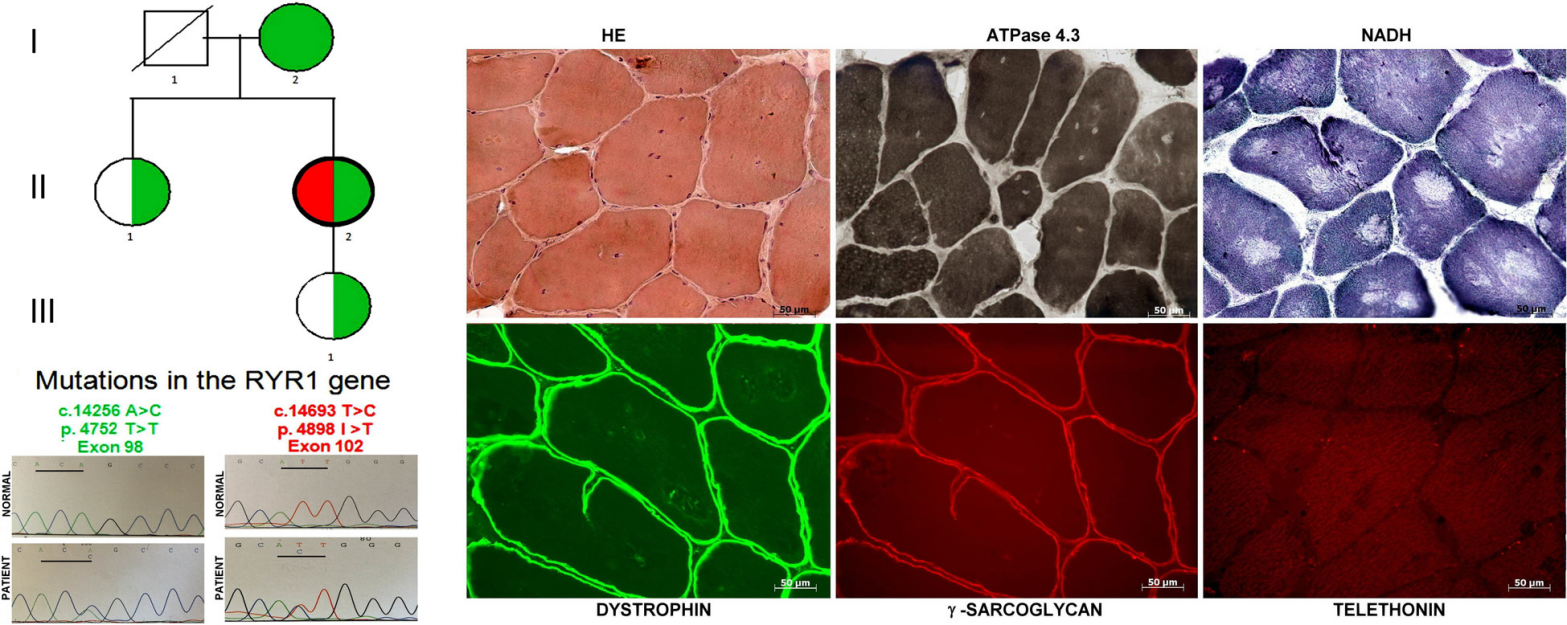
**The pedigree of the family, and mutations identified in each member of the family, and respective eletroferogram of part of exon 102 and exon 98 of the *RYR1 *gene illustrating the missense alterations.** Histological reactions showing HE, ATPase 4.3 and NADH staining, and immunohistochemical analysis for dystrophin, γ-SG and telethonin. (Magnification – X200).

The index case, a 48-year-old Caucasian female, was referred to our service with a history of muscle weakness since childhood. Her delivery and peri-natal period were uneventful. She was a hypotonic baby, had delayed milestones and presented congenital dislocation of the hip as a child. Her present clinical evaluation consisted of mild facial and proximal muscle weakness, lumbar lordosis and scoliosis. She had no cardiac abnormalities and no respiratory complaints. An electromyography exam done in her childhood showed a myopathic pattern. Serum creatine kinase activity (CK) was normal.A muscle biopsy of biceps brachii was performed under local anesthesia. Histological analysis of hematoxilin/eosin (H&E) staining revealed variation in fiber size, split fibers, some fibers with membrane debris in its interior and presence of multiple nuclei within the majority of fibers. Myofibrillar ATPase reaction showed a predominance of type I fibers, and NADH-adenine dinucleotide-tetrazolium reductase reaction revealed areas devoid of oxidative activity (cores) in almost all fibers (Figure 1). Normal localization and distribution was found for all studied muscle proteins: dystrophin, the 4 sarcoglycan, dysferlin, calpain 3, telethonin, and α2-laminin, excluding several forms of limb-girdle muscular dystrophies.

For molecular analysis, Sanger sequencing for the C-terminal region (exons 94 to 106) of the *RYR1* gene was first done in genomic DNA, extracted from peripheral blood lymphocytes [16], using PCR amplification with described primers [17]. Sequencing was done using sequencing kit (Applied Biosystems®) on an ABI 3730 automated DNA sequencer (Life Technologies).

Subsequently, next generation sequencing of the whole *RYR1* gene was done using Illumina’s Nextera kits for library preparation and custom capture of 88 genes involved in neuromuscular disorders. Sequencing was performed at the Illumina MiSeq sequencer, and variants were filtered and compared to the control populations of 1000 Genomes, NIH, and 6500 Exome Sequencing Project from Washington University.

Molecular analysis of the *RYR1* gene showed two different mutations in the patient (II-2): a missense mutation c.14256 A > C in exon 98, leading to a silencing alteration p.4752 T > T, and a pathogenic missense mutation c.14693 T > C in exon 102, which results in the p.4898 I > T substitution in the protein. The c.14256 A > C mutation was also identified in her daughter (III-1). To exclude a possible unknown deleterious potential, we studied the mother (I-2) and sister (II-1) of the index case, and the same alteration was found in both: in a homozygous state in the mother, and heterozygous state in the sister (Figure 1). None of the relatives presented the pathogenic mutation in exon 102. Additional next generation sequencing study of the whole *RYR1* gene in the patients revealed, in addition to the c.14693 T > C and c.14256 A > C mutations, the presence of 5 common silent polymorphisms in homozygosis and 8 polymorphisms in heterozygosis (Table 1).

**Table 1:** **Variants identified in the *RYR1 *** **gene trough NGS sequencing of a panel of 88 muscle genes using MiSeq – Ilumina panel**

**AAChange.refGene**	**6500 exomes**	**1000 genomes**
Homozygouse variants		
RYR1:NM_000540:exon7:c.A594G:p.L198L,RYR1:NM_001042723:exon7:c.A594G:p.L198L	0,603029	0,6
RYR1:NM_000540:exon11:c.T1077C:p.A359A,RYR1:NM_001042723:exon11:c.T1077C:p.A359A	0,895202	0,93
RYR1:NM_000540:exon15:c.G1668A:p.S556S,RYR1:NM_001042723:exon15:c.G1668A:p.S556S	0,632093	0,64
RYR1:NM_000540:exon19:c.C2286T:p.P762P,RYR1:NM_001042723:exon19:c.C2286T:p.P762P	0,617023	0,62
RYR1:NM_000540:exon24:c.G2943A:p.T981T,RYR1:NM_001042723:exon24:c.G2943A:p.T981T	0,597092	0,6
Heterozygouse variants		
RYR1:NM_000540:exon45:c.C7260T:p.H2420H,RYR1:NM_001042723:exon45:c.C7260T:p.H2420H	0,02914	0,03
RYR1:NM_000540:exon49:c.C7872T:p.R2624R,RYR1:NM_001042723:exon49:c.C7872T:p.R2624R	0,168538	0,16
RYR1:NM_000540:exon50:c.G7977A:p.T2659T,RYR1:NM_001042723:exon50:c.G7977A:p.T2659T	0,319545	0,36
RYR1:NM_000540:exon51:c.T8118C:p.I2706I,RYR1:NM_001042723:exon51:c.T8118C:p.I2706I	0,319775	0,36
RYR1:NM_000540:exon51:c.T8190C:p.D2730D,RYR1:NM_001042723:exon51:c.T8190C:p.D2730D	0,32362	0,37
RYR1:NM_000540:exon53:c.G8337A:p.E2779E,RYR1:NM_001042723:exon53:c.G8337A:p.E2779E	0,308213	0,35
RYR1:NM_000540:exon55:c.T8589C:p.S2863S,RYR1:NM_001042723:exon55:c.T8589C:p.S2863S	0,313624	0,36
RYR1:NM_000540:exon62:c.A9186G:p.P3062P,RYR1:NM_001042723:exon62:c.A9186G:p.P3062P	0,310857	0,35
RYR1:NM_001042723:exon97:c.A14241C:p.T4747T,RYR1:NM_000540:exon98:c.A14256C:p.T4752T	0,108642	0,14
RYR1:NM_001042723:exon101:c.T14678C:p.I4893T,RYR1:NM_000540:exon102:c.T14693C:p.I4898T	0	0

NM_: Accession prefix for RNA and protein products that are mainly derived from GenBank cDNA and EST data and are supported by the RefSeq eukaryotic curation group. http://www.ncbi.nlm.nih.gov/refseq/about/.

Frequency in the 6500 exomes and1000 genomes.

## Discussion

Central core myopathy (CCD) is one of the most common described congenital myopathies, yet its prevalence and incidence are still uncertain. A regional study in England demonstrated a frequency for CCD of 1 in 250.000 individuals [17], while another study in Japan revealed that carrier frequency for heterozygous *RYR1* mutations in that population could be as high as 1 in 2000 [13].

Most patients with RYR-related CCD usually have mild or moderate axial and proximal weakness marked in the hip girdle, characterized generally in infancy with hypotonia or in early childhood with motor developmental delay [3]. Skeletal muscle complications such as congenital developmental hip dysplasia, scoliosis and foot deformities (talipes equinovarus) are quite common, as well as myalgia, muscle stiffness, and exertional weakness with or without rhabdomyolysis are frequently related and may be the only exhibiting features. Bulbar and respiratory muscle involvement is unusual, but has been seen in the most severe neonatal cases, and sporadically in adults. Still, there can be significant variability in gravity and age of onset of CCD and marked phenotype heterogeneity, frequently within the same family, has been documented [18-20].

Our patient presented clinical features suggesting a congenital myopathy with a relatively mild progression. Histological analysis confirmed the diagnosis of CCD. Although neither her parents, sister nor daughter presented similar symptoms, a possible late-onset manifestation, or a very mild weakness raised doubt about the genetic counseling in this family. The youngest patient’s daughter could have inherited the mutations and was still in her reproductive years.

The initial molecular analysis encompassing the C-terminal region of the *RYR1* gene revealed two different mutations in the patient; one found in exon 102, which was already described as pathogenic [10], whereas the mutation in exon 98 has been described as a single nucleotide polymorphism, with allele frequency between 0,87/0,13 and 0,95/0,05, and heterozygosis of 0,22 [21-23]. The c.14693 T > C mutation in exon 102 has been first described in a large Mexican affected family [10]. Twenty-one individuals of 35 members were diagnosed with CCD after clinical evaluation, and symptoms ranged from mild to severe, associating this mutation with a highly variable phenotype of CCD. Affected individuals presented with at least one of the symptoms consistent with CCD: foetal hypotonia, muscular weakness at birth, delayed motor milestones, proximal muscle weakness, foot deformities, scoliosis and congenital hip dislocation [10]. Subsequently, this same mutation was also described in other populations. It was found in 3 patients from Italian and Swiss-Italian families, with muscle weakness as the main symptom [24] and in two other families from France and North America, in which both presented muscular weakness and delayed motor milestones [15]. In Japan, one patient was identified, with very severe clinical condition, including decreased foetal movements, hypotonia, respiratory insufficiency at birth, delayed milestones, congenital dislocation of the hip, scoliosis and facial muscle weakness [13]. Considering the above clinical descriptions, the c.14693 T > C mutation is compatible both with severe and mild phenotypes.

As our patient is presenting a relatively benign clinical course, probably there is no significant association between her pathogenic mutation and the p.4752 T > T synonymous polymorphism, nor with any of the other silencing mutations later identified in her DNA, that could be acting as a modifier of her phenotype, by affecting transcription, splicing, mRNA transport, and translation.

## Conclusions

As our patient’s phenotype is within the range of other patients described with the same heterozygous mutation, the associated polymorphisms identified in her DNA apparently are not contributing to worsen her phenotype. Additionally, since all the tested relatives have only the p.4752 T > T polymorphism and don’t present any typical CCD features, it can be inferred that this variant alone, in a homozygous or heterozygous state is not responsible for the disease.

The case described above illustrates the present reality where new methods for wide genome screening are becoming more accessible and able to identify a great variety of mutations and polymorphisms in patients and their families. It is still impossible to tell if this information helps making more diagnosis and genotype/phenotype associations, or if it turns the diagnostic process more confusing and complex. It is important to always have this in mind before reaching to a definite diagnosis for patients and involved family.

## Consent

Written informed consent was obtained from the patient, her daughter, mother and sister, for genetic testing and for publication of this Case Report including any accompanying images. A copy of the written consent is available for review by the Editor-in-Chief of this journal. This study was approved by the Comissão Nacional de Ética em Pesquisa (CONEP - Brazilian ethics committee) – University of Sao Paulo and Federal University of Sao Paulo, under the number 297.008.

## Abbreviations

CCD: Central core disease; CK: Creatine kinase; H&E: Hematoxilin/eosin; MH: Malignant hyperthermia; NADH: Adenine dinucleotide-tetrazolium reductase; NGS: Next generation sequencing; *RYR1*: Ryanodin receptor gene 1.

## Competing interests

The authors declare that they have no competing interests.

## Authors’ contributions

Conception and design: MV, TC, and ASBO. Acquisition of data: TC, SA, NCVL, LUY, HCS, GLY, RCP, Analysis and interpretation of data: TC, MZ, and MV. Involved in drafting the manuscript or revising it critically for important intellectual content: TC, HCS, and MV. Gave final approval of the version to be published: MV and TC. All authors read and approved the final manuscript.
